# Pathophysiological mechanisms of organ injury in Fabry disease: Update via multi-omics

**DOI:** 10.1016/j.gendis.2025.101949

**Published:** 2025-11-25

**Authors:** Zhiyuan Wei, Junlan Yang, Zhongyu Han, Xiaoliang Zhang, Bin Wang

**Affiliations:** a Department of Nephrology, Zhong Da Hospital, Southeast University, Nanjing, Jiangsu 210009, China; b Southeast University School of Medicine, Nanjing, Jiangsu 210009, China

**Keywords:** Fabry disease, Omics, Organ injury, Pathophysiological mechanisms, Therapy

## Abstract

Fabry disease (FD) is an X-linked lysosomal storage disorder caused by mutations in *GLA* gene, which result in deficient α-galactosidase A activity, leading to intralysosomal accumulation of metabolic substrates and multi-organ injury. Due to the heterogeneity of clinical phenotypes and limitations of current diagnostic modalities, the diagnosis of FD remains challenging. Enzyme replacement therapy is the cornerstone of FD treatment. However, this therapy cannot fully reverse pre-existing organ damage, and patients will still face an unfavorable prognosis. The mechanisms of organ injury in FD cannot fully explain reduced enzyme activity alone and therefore warrant further elucidation. In recent years, omics, including transcriptomics, proteomics, and metabolomics, have demonstrated great potential for elucidating FD pathophysiology, identifying novel biomarkers, and uncovering therapeutic targets. This review presents the pathophysiological mechanisms of FD in its principal target organs and summarizes recent advances in omics applied to its primary target organs, chiefly the kidney and heart. Omics-based investigations hold promise for advancing precision medicine in FD, offering new avenues for early diagnosis and personalized therapy.

## Introduction

Fabry disease (FD, OMIM #301500) is an X-linked inherited disorder caused by mutations in the *GLA* gene (Xq22.1), which cause deficient α-galactosidase A (α-Gal A) activity, leading to intralysosomal accumulation of metabolic substrates, primarily globotriaosylceramide (Gb3) and globotriaosylsphingosine (Lyso-Gb3), resulting in lysosomal dysfunction.^1^ Based on clinical features and α-Gal A activity, FD can be classified into classic phenotype and late-onset phenotype. Classic FD predominantly affects males and initially presents with acroparesthesias and burning sensations in the limbs, accompanied by angiokeratomas, characterized by red to purple papular lesions on the trunk, limbs, and face. Subsequently, patients develop progressive dysfunction of the heart, kidneys, nervous system, eyes, and ears, ultimately leading to life-threatening complications, such as end-stage renal failure, heart failure, serious arrhythmias, and stroke.^2^ Late-onset FD is more frequently seen in females, who retain residual α-Gal A activity. Their clinical manifestations appear later in life, which are often atypical and typically present with isolated organ involvement, most frequently the heart or the kidneys.

The diagnosis of FD is complex and requires integration of clinical symptoms, family history, α-Gal A activity, quantification of Gb3 in plasma and in urine, genetic testing, and laboratory investigations of target organs. Although epidemiological studies estimate the prevalence of classic FD at approximately 1 in 40,000 to 1 in 170,000, the actual prevalence may be higher.^3^ Several factors contribute to challenges in the diagnosis of FD. Firstly, as a multi-organ-involved disease, FD exhibits marked clinical heterogeneity, and the pathophysiological mechanisms of target organs remain incompletely understood. Furthermore, because of the selective inactivation of the X chromosome, heterozygous females cannot be accurately diagnosed by α-Gal A activity alone. Moreover, while genetic testing is the most definitive diagnostic tool, its high cost and the complexity of mutation interpretation cannot be overlooked. Biomarkers such as Lyso-Gb3 can aid in disease assessment, but the levels of these biomarkers are not uniformly elevated across all patients and do not consistently correlate with the severity of organ involvement.^4^ Additionally, laboratory tests of target organs, such as late gadolinium enhancement on cardiac MRI, can identify organ injury but lack sensitivity for early detection of FD.^5^ Our team^6^ has demonstrated that myeloid bodies in urinary sediment serve as biomarkers for the early diagnosis and monitoring of the efficacy of enzyme replacement therapy (ERT) of FD, but these findings require further validation.

The challenges in diagnosing FD highlight the need to develop novel diagnostic strategies, which require a deeper understanding of the pathophysiology of FD. In recent years, omics, including transcriptomics, proteomics, and metabolomics, have advanced rapidly. Omics can identify specific molecular biomarkers, uncover dysregulated signaling pathways, and reveal potential therapeutic targets. Consequently, omics have been broadly applied to elucidate mechanisms of diseases, develop innovative diagnostic methods, enable personalized medicine, and accelerate drug development. This review summarizes current insights into the pathophysiological mechanisms driving organ injury in FD and explores the application of omics in this context, thereby providing a theoretical foundation for the development of diagnostics and therapeutics for FD.

## Introduction to omics and its use in Fabry disease

**Figure 1:**
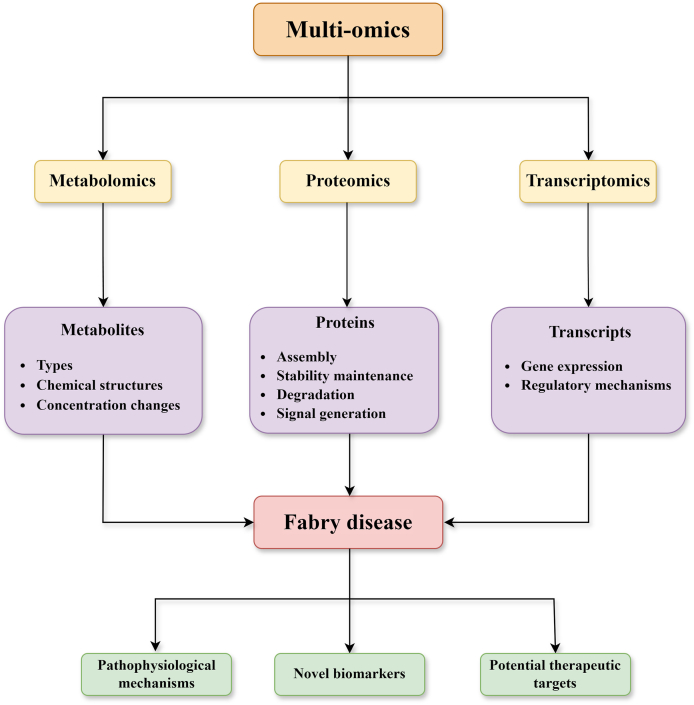
Applications and significance of omics in Fabry disease. Transcriptomics, proteomics, and metabolomics have been applied in Fabry disease-related research. Transcriptomics aims to delineate gene expression and its regulatory mechanisms of physiological and pathological states by profiling all transcripts. Proteomics offers insights into the mechanisms of protein assembly, stability maintenance, degradation, and signal transduction. Metabolomics can analyze the types, chemical structures, and concentration changes of metabolites within the body. Omics have been applied to elucidate pathophysiological mechanisms, find novel biomarkers, and identify potential therapeutic targets of Fabry disease.

In the context of an urgent need for novel technologies to elucidate the pathophysiology of FD, omics have proven indispensable. Omics are approaches using high-throughput technologies, information technologies, and systems biology to perform comprehensive analyses of biomolecular information. Transcriptomics, proteomics, and metabolomics have been applied in FD-related research, offering powerful tools and new perspectives for in-depth investigation of FD (Fig. 1).

Transcriptomics aims to delineate gene expression and its regulatory mechanisms of physiological and pathological states by profiling all transcripts. Seemann et al^7^ demonstrated that proteostasis regulators, such as MG132, bortezomib, Clasto-Lactacystin β-lactone, and Eeyarestatin I, can inhibit proteasomal degradation and up-regulate *GLA* expression, thereby enhancing α-Gal A activity and mitigating Gb3 accumulation. Moreover, both bortezomib and MG132 were shown to modulate the expression of genes involved in endoplasmic reticulum-associated degradation arising from *GLA* missense mutations. These findings underscore a mechanistic link between proteostasis regulators and the pathophysiology of FD. More recently, RNA sequencing has been applied in FD research to identify disease-specific gene expression signatures and to evaluate responses to therapy. For instance, Delaleu et al^8^ conducted a transcriptomic analysis of Fabry nephropathy in patients before and after ERT, which revealed that while early ERT can normalize many dysregulated genes, certain pathways remain persistently altered. These ERT-resistant gene expression changes point to potential biomarkers for monitoring disease course and suggest additional therapeutic targets for adjunct treatment.

Proteomics offers insights into the mechanisms of protein assembly, stability, degradation, and signal transduction. Proteomics can rapidly and sensitively identify multiple specific biomarkers and facilitate the discovery of novel pathogenic mechanisms and their translation into clinical diagnostics in FD patients. In recent years, there has been a growing number of proteomics studies in FD, most of which focus on profiling differentially expressed proteins in patients and elucidating associated pathogenic pathways. A summary of the applications of proteomics in FD research is provided in Table 1.^9^, ^10^, ^11^, ^12^, ^13^, ^14^, ^15^, ^16^, ^17^, ^18^, ^19^, ^20^, ^21^, ^22^, ^23^, ^24^, ^25^, ^26^, ^27^

**Table 1:** Studies of proteomics in Fabry disease.

Subjects	Samples	Conclusion	Researchers	Year	References
1 FD patient and 2 healthy controls	iPSC	Organ dysfunction cannot be fully explained by Gb3 accumulation alone in FD	Ryan et al	2025	9
2 FD patients and 2 healthy controls	iPSC	Ferroptosis is a potential pathway in FD cell pathology	Wise et al	2024	10
50 FD patients and 50 healthy controls	Plasma	APOA4, FETUA, and APOC3 were associated with the presence of complications in FD	López-Valverde et al	2024	11
FD rats and WT rats	Schwann cell	S100A10 (p11) is the most highly up-regulated protein in Fabry-SCM	Waltz et al	2024	12
55 FD patients and 30 healthy controls	Plasma	615 differentially expressed proteins were associated with tissue-specific remodeling	Tebani et al	2023	13
Fabry clones and WT clones	Podocytes	α-synuclein (SNCA) accumulation was identified as a key event mediating podocyte injury	Braun et al	2023	14
8 GLA^*-*^ zebrafish and 8 WT zebrafish	Renal tissues	639 proteins were differentially expressed; 527 proteins were down-regulated and 112 up-regulated	Elsaid et al	2023	15
Fabry clones and WT clones	Podocytes	Loss of α-Gal A function results in profound changes in cellular pathways	Jehn et al	2021	16
69 FD patients and 83 healthy controls	Plasma	4 proteins (FGF2, VEGFA, VEGFC and IL-7) were differentially expressed between the 2 groups	Tebani et al	2020	17
2 FD patients and 4 healthy controls	iPSC	Accumulation of the lysosomal protein LIMP-2 and secretion of cathepsin F and HSPA2/HSP70-2	Birket et al	2019	18
Fabry clones and WT clones	Cardiomyocytes	The Rab GTPases involved in exocytotic vesicle release were significantly downregulated	Song et al	2019	19
72 FD patients and 72 healthy controls	Urine	95 peptide biomarkers revealed, 50 showed significant correlations with clinical parameters	Weidemann et al	2019	20
15 FD patients	Renal tissues	Classic and late onset cases differed significantly rather than males and females	L'Imperio et al	2019	21
66 FD patients and 10 healthy controls	Urine	6 urinary proteins were elevated in the early-stage/asymptomatic Fabry group	Doykov et al	2019	22
7 FD patients and 7 healthy controls	Urine	Indicating an involvement of the coordinated lysosomal expression and regulation network	Slaats et al	2018	23
32 FD patients and 14 healthy controls	Plasma	Identified an 8-protein panel for male FD patients and a 9-protein panel for female patients	Hollander et al	2015	24
23 FD patients and 12 healthy controls	Urine	Up-regulation of uromodulin, prostaglandin H2 d-isomerase, and prosaposin was demonstrated	Matafora et al	2015	25
10 FD patients and 10 healthy controls	Urine	Prosaposin and GM2 activator protein (GM2AP) were elevated in patient groups	Manwaring et al	2013	26
20 FD patients and 10 healthy controls	Urine	The concentration of some proteins was more than three times higher in the FD samples	Vojtová et al	2010	27

Note: FD, Fabry disease; iPSC, induced pluripotent stem cell; LVH, left ventricular hypertrophy; WT, wild type; SCM, Schwann cell-conditioned media; ERT, enzyme replacement therapy.

Metabolite levels can fluctuate rapidly in response to physiological changes, often more quickly than changes at the protein level.^4^ Metabolomics has been widely applied due to its superior separation efficiency, high sensitivity, specificity, and analytical throughput. Important metabolomic studies in FD are summarized in Table 2.^28^, ^29^, ^30^, ^31^, ^32^, ^33^, ^34^, ^35^, ^36^ To date, the metabolites definitively confirmed in FD are all Gb3 analogues. However, the correlation between Gb3 levels and disease severity is often weak or variable, limiting their reliability as biomarkers for therapeutic monitoring.^37^ Kim et al^29^ explored metabolites beyond Gb3, identifying 27 and 23 differentially expressed metabolites in the serum and urine of the Fabry mouse model, respectively. In serum, increases were observed in glutathione, glutathione disulfide, citrulline, and kynurenine, which are involved in oxidative stress, nitric oxide biosynthesis, and inflammation. Meanwhile, urinary profiles revealed alterations in metabolites related to pyruvate metabolism, tyrosine metabolism, and the tricarboxylic acid cycle.

**Table 2:** Studies of metabolomics in Fabry disease.

Subjects	Samples	Conclusion	Researchers	Year	References
6 FD patients and matching healthy people	iPSC	Distinct Gb_3_ isoform profiles and delayed relaxation consistent with diastolic dysfunction	Nicholls et al	2025	28
α-Gal A knockout mice and WT mice	Serum and urine	Altered metabolic signatures associated with oxidative stress, inflammation, nitric oxide biosynthesis, and immune regulation	Kim et al	2024	29
15 FD patients and 13 healthy people	Plasma	The systemic changes in FD are reflected in the plasma and platelet lipidome	Burla et al	2024	30
*GLA*^*-*^ mice and WT mice	Plasma	An mRNA-based therapeutic agent can affect levels of metabolites	Zhang et al	2024	31
A suspected FD patient and 2 healthy people	Renal tissue	A Ga2-related lipid biomarker was substantially higher in the patient's renal tissue biopsy	Yazd et al	2021	32
66 FD patients and 60 healthy people	Plasma	Identified 86 metabolites that are differentially expressed	Ducatez et al	2021	33
68 FD patients and 40 healthy people	Plasma	Plasma levels of inflammatory biomarkers and cardiac remodeling biomarkers are elevated	Yogasundaram et al	2018	34
150 FD patients and 95 healthy people	Urine	5 patients with the late-onset cardiac mutation p.N215S showed abnormal concentrations of methylated Gb3 isoforms	Abaoui et al	2016	35
63 FD patients and 59 healthy people	Urine	7 novel Fabry biomarkers were found in urine	Auray-Blais et al	2012	36

Note: WT, wild type; FD, Fabry disease; iPSC, induced pluripotent stem cell; Gb3, globotriaosylceramide; Lyso-Gb3, globotriaosylsphingosine.

As a rare disease, FD research is constrained by relatively small sample sizes. Therefore, the findings from omics-based studies in FD warrant validation in larger patient cohorts.

## Advances in the pathophysiology of target organs in Fabry disease

**Figure 2:**
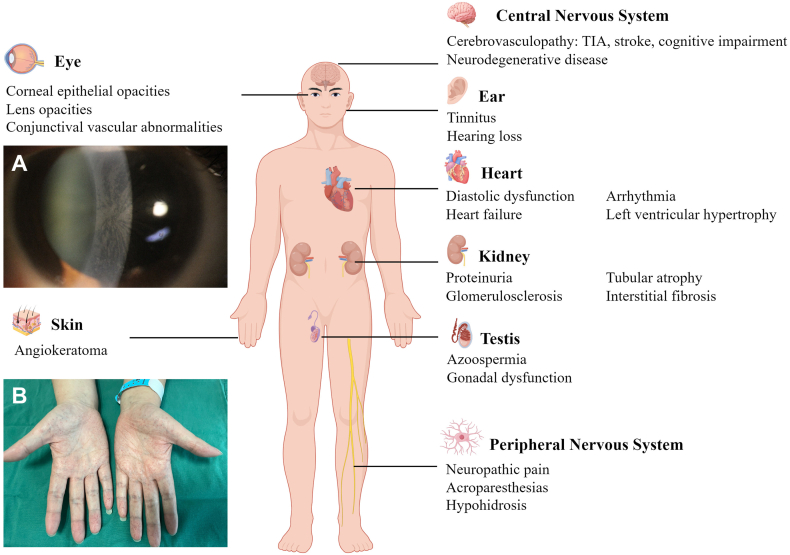
Manifestations of organ injury in Fabry disease. Fabry disease can cause multi-organ damage. Renal involvement is characterized principally by proteinuria and structural alterations, including glomerulosclerosis, tubular atrophy, and interstitial fibrosis, ultimately progressing to end-stage renal failure. Cardiac involvement predominantly presents as left ventricular hypertrophy, diastolic dysfunction, myocardial fibrosis, arrhythmias, and heart failure. Cerebrovascular injuries can result in transient ischemic attacks, ischemic stroke, and vascular cognitive impairment. Autonomic disturbances include neuropathic pain, acroparesthesias, and hypohidrosis. Gb3 accumulation in ocular tissues can cause conjunctival and retinal vascular tortuosity, cataracts, and cornea verticillate **(A)**. Cornea verticillata is a whorl-like epithelial opacity of the cornea observed in Fabry disease. Hearing loss, sudden deafness, and tinnitus are the main manifestations of auditory involvement. Cutaneous manifestations are often the earliest clinical signs, most notably the formation of angiokeratomas **(B)**. Angiokeratomas are dark red to purple papules caused by capillary dilatation with epidermal thickening in Fabry disease. Reproductive system involvement is characterized by azoospermia and gonadal dysfunction. The patient images in (A) and (B) are from Southeast University Zhongda Hospital (published with patient consent). This figure is drawn by Figdraw (www.figdraw.com).

To date, over 1000 GLA variants have been catalogued in the Human Gene Mutation Database (HGMD), the most of which are missense mutations, accounting for approximately 69% (http://www.hgmd.cf.ac.uk/ac/gene.php gene = GLA; accessed 22 September 2025). The *GLA* gene, which spans 14 kb at Xq22.1, encodes α-Gal A.^38^ α-Gal A is a 429-amino-acid polypeptide that functions as an enzyme involved in the catabolism of glycosphingolipids. α-Gal A deficiency prevents degradation of glycosphingolipids at the cell membrane, leading to their accumulation in organs, including the kidneys, heart, and nervous system,^39^^,^^40^ resulting in the diverse clinical manifestations (Fig. 2).

## Kidney

**Figure 3:**
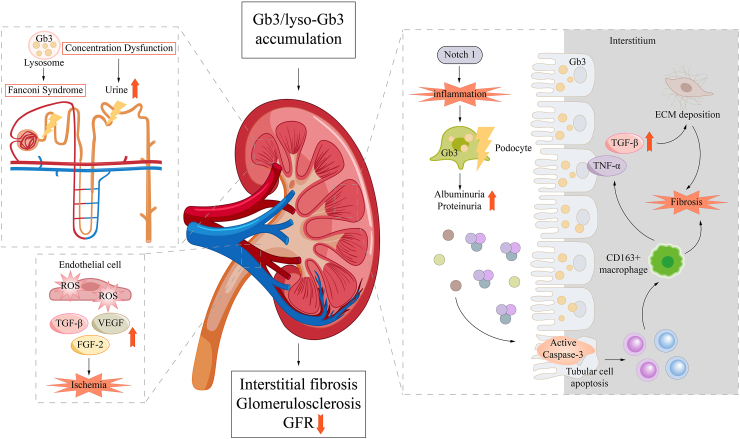
Mechanisms of renal injury in Fabry disease. Gb3 was deposited in podocytes and interfered with cell function. Notch 1 mediates inflammatory and fibrotic responses, while Gb3 deposition leads to foot process fusion, decreased expression of slit membrane proteins such as nephrin, and proteinuria formation. Gb3 disrupts lysosomal membrane stability, leading to enzyme leakage and activation of apoptotic pathways. TNF-α enhances the inflammatory response and synergizes with TGF-β to promote fibrosis. Macrophage infiltration (CD163^+^) accelerates the process of fibrosis. ROS damage DNA, proteins, and lipids and promote the release of inflammatory factors such as TNF-α. Eventually, renal interstitial fibrosis, glomerulosclerosis, and decreased glomerular filtration rate occur.

Renal involvement in FD is characterized principally by proteinuria and structural alterations, including glomerulosclerosis, tubular atrophy, and interstitial fibrosis,^2^ ultimately progressing to end-stage renal failure. In the kidney, accumulated metabolic substrates induce inflammation, oxidative stress, and tissue fibrosis through up-regulation of transforming growth factor-β1 (TGF-β1) expression, increased CD47 production, enhanced reactive oxygen species (ROS) generation and adhesion molecule expression, and induction of epithelial-to-mesenchymal transition,^41^, ^42^, ^43^ culminating in podocyte injury (Fig. 3). Podocytes are a critical component of the glomerular filtration barrier, responsible for maintaining structural integrity of glomerular filtration barrier, facilitating intraglomerular signaling, and preventing clogging of glomerular filtration barrier.^44^ Snanoudj et al^45^ performed transcriptomic profiling on the FD podocyte cell line, identifying 247 differentially expressed genes. These genes are closely linked to pathways of oxidative stress, inflammation, fatty-acid metabolism, collagen and extracellular-matrix homeostasis, apoptosis, and autophagy, providing comprehensive molecular insights into podocyte dysfunction in FD.

Beyond metabolic substrate accumulation, other factors also contribute to renal injury in FD. Kim et al^46^ demonstrated that glutathione metabolism was reduced in *GLA*-mutant human renal organoids. Glutathione replacement therapy can alleviate oxidative stress, mitigate renal structural abnormalities, and decrease apoptosis, indicating that impaired glutathione metabolism represents a potential mechanism underlying renal injury in FD. Turkmen et al^47^ uncovered the potential role for immune cells. Compared with healthy controls, FD patients exhibited significantly increased infiltration of B cells and plasma cells, but their mechanisms remain to be elucidated. Additionally, Laffer et al^48^ reported pronounced activation of the complement system in FD nephropathy. Serum C3a concentrations are increased thirty times, and ERT does not normalize these levels. Together, these studies confirm the existence of novel mechanisms in FD nephropathy beyond metabolic substrate accumulation.

Omics have provided new perspectives for investigating the mechanisms of renal pathology in FD. Using proteomics, Ryan et al^9^ focused on the longitudinal analyses in patient-induced pluripotent stem cell (iPSC) podocytes, finding progressive lysosomal stress with sphingolipid pathway remodeling, indicating sustained organelle dysfunction that is not fully explained by Gb3 accumulation alone. In agreement with this, Wise et al^10^ found that iPSC-derived podocytes from Fabry patients implicate ALOX15-driven ferroptosis as a key injury mechanism, highlighting lipid peroxidation and iron-dependent cell death as potential therapeutic targets. Similarly, Braun et al^14^ identified α-synuclein (SNCA) accumulation as an independent key mediator of podocyte injury in FD nephropathy. Elsaid et al^15^^,^^49^ found down-regulation of lysosomal and mitochondrial proteins as well as oxidative phosphorylation pathways in renal tissue of *GLA*-knockout zebrafish. Meanwhile, energy-related pathways, including carbon, glycolysis, and galactose metabolism, were disturbed. The investigators also observed abnormal mitochondrial shape, disrupted cristae morphology, altered mitochondrial volume, and lower antioxidant activity in *GLA*-knockout zebrafish. Collectively, these omics-based studies have uncovered novel pathophysiological mechanisms of renal damage in FD.

## Cardiovascular system

**Figure 4:**
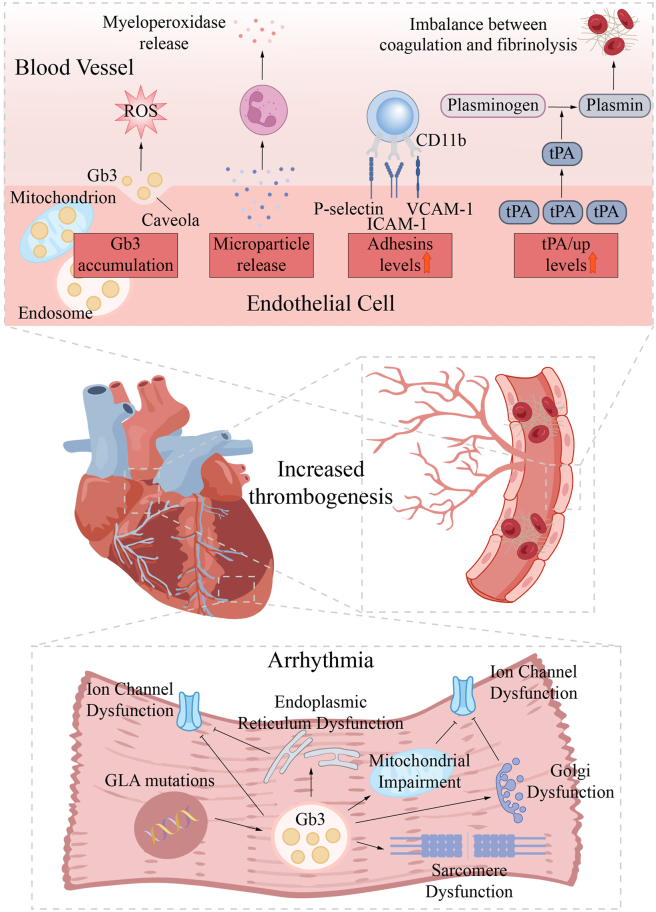
Mechanisms of cardiac injury in Fabry disease. The accumulation of Gb3 in endothelial cells directly impairs cellular function and triggers an inflammatory and procoagulant state. Gb3 deposition leads to disturbance of the fibrinolytic system and promotes thrombosis. Chronic inflammation and ischemia eventually lead to collagen deposition, myocardial stiffness, and impaired diastolic function. Gb3 deposition leads to mitochondrial dysfunction and reduced ATP production, triggering an energy crisis in cardiomyocytes. Endoplasmic reticulum and Golgi apparatus dysfunction and abnormal protein folding and trafficking activate unfolded protein response (UPR) and promote cell apoptosis. Gb3 deposition interferes with ion channel function, leading to abnormal action potentials. The structure of contractile proteins in cardiomyocytes is abnormal, which aggravates cardiac insufficiency.

Cardiac involvement in FD predominantly presents as left ventricular hypertrophy, diastolic dysfunction, myocardial fibrosis, arrhythmias, and heart failure, whereas coronary artery disease and systolic dysfunction are uncommon.^50^ In early stages, pathological changes are often localized to the atrioventricular node, resulting in conduction abnormalities,^51^ and subsequently extend to cardiomyocytes. Gb3 accumulation disrupts autophagy, resulting in increased mitochondrial ROS generation and apoptosis. Concurrently, Gb3 deposition triggers inflammatory responses, causing endothelial dysfunction, myocardial remodeling, and fibrosis of the heart^52^^,^^53^ (Fig. 4).

Oxidative stress plays a critical role in the development of cardiac injury in FD. Salamon et al^54^ demonstrated that miR-184 was independently associated with cardiac injury in FD patients. This association is mediated by ROS-induced oxidation-dependent modification of miR-184, which down-regulates Bcl-xL and Bcl-w, thereby promoting apoptosis. Furthermore, miR-184 levels are modulated by ERT and may serve as biomarkers for assessing both the severity of cardiac involvement and the efficacy of ERT. Shen et al^55^ identified tetrahydrobiopterin (BH4) deficiency in myocardial biopsies from FD patients. BH4 levels were inversely correlated with Gb3, and ERT only partially restored BH4 deficiency. As a cofactor related to cellular antioxidant defense and NOS uncoupling, BH4 plays a critical role in the oxidative stress process. These findings suggest that cardiac injury in FD may involve mechanisms beyond Gb3 accumulation, or that ERT has limited efficacy in reversing myocardial damage caused by substrate deposition. Similarly, Frustaci et al^56^^,^^57^ demonstrated that myocardial damage in FD could not be fully reversed by ERT even in early disease stages, owing to down-regulation of the myocardial mannose-6-phosphate receptor (M6Pr). M6Pr mediates cardiomyocyte uptake and internalization, representing a novel therapeutic target for FD cardiac injury.

Omics have also been extensively applied to investigate cardiac injury in FD. Song et al^19^ utilized proteomics to reveal a marked down-regulation of proteins involved in cytoskeletal dynamics and extracellular vesicle secretion. Rab GTPase-mediated vesicle secretion was implicated as a potential driver of cardiomyocyte hypertrophy in FD. Burla et al^30^ conducted lipidomic profiling and identified differential expression of plasma lipids, including lyso-dihexosylceramides, sphingoid base 1-phosphates (S1P), and GM3 ganglioside. Furthermore, FD patients exhibited accumulation of acylcarnitines, C16:0 sphingolipids, and S1P in platelets. These findings indicate that lipidomic alterations in plasma and platelets extend beyond those caused by substrate accumulation and suggest a potential role for platelets in FD cardiac disease. Additionally, Kobayashi et al^58^ employed cap analysis of gene expression (CAGE) on cardiac injury in FD and observed significant up-regulation of chimerin 1 (CHN1). CHN1 is associated with oxidative stress and myocardial fibrosis and may serve as a potential biomarker for FD cardiac involvement.

## Nervous system

**Figure 5:**
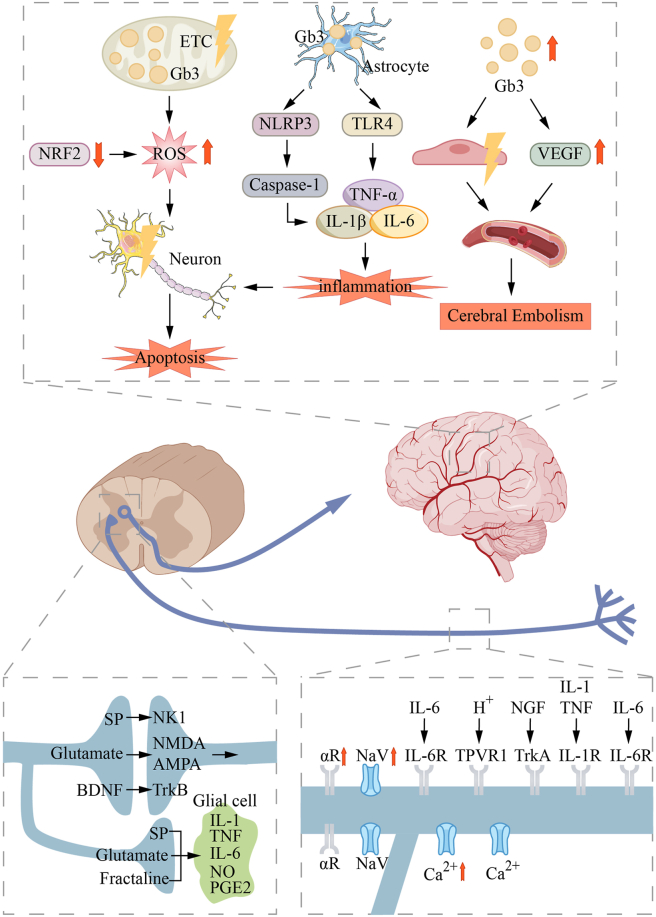
Mechanisms of nervous system injury in Fabry disease. Gb3 accumulation induces mitochondrial dysfunction, leading to ROS overproduction and triggering oxidative damage. Continuous oxidative stress may inhibit NRF2 activity and exacerbate neuronal damage. The NLRP3 inflammasome is activated by ROS and Gb3, leading to Caspase-1 cleavage and release of proinflammatory factors. TNF-α, IL-1β, and IL-6 are secreted by microglia and astrocytes, amplifying neuroinflammation and disrupting the blood–brain barrier. Inflammation and oxidative stress lead to neuronal apoptosis. Gb3 acts on the vascular endothelium, and VEGF-mediated vascular permeability increases, which may contribute to the risk of cerebral embolism. α2 adrenergic receptor, which may be dysregulated in Fabry patients, leads to abnormal sympathetic activation and exacerbates neuropathic pain. The up-regulation of voltage-gated sodium channels (NaV) enhances action potential firing, resulting in spontaneous pain. Inflammation and glial activation lead to excessive release of NGF, which binds to the TrkA receptor, up-regulates thermal pain receptor (TRPV1) and NaV channels, and enhances thermal and mechanical pain sensitivity.

Neurological involvement in FD can be categorized into central nervous system manifestations, predominantly characterized by cerebrovascular lesions, and peripheral nervous system manifestations, which present with limb pain, abnormal sweating, and altered temperature sensation (Fig. 5), and extrapyramidal involvement has also been reported occasionally.^59^ Cerebrovascular lesions in FD are primarily associated with glycosphingolipid accumulation in cerebral vessels, secondary inflammatory responses, and vascular wall thickening and thrombosis resulting from endothelial dysfunction. These vascular injuries can result in transient ischemic attacks, ischemic stroke, and vascular cognitive impairment.^60^ Moreover, glycosphingolipids accumulate not only in cerebral vasculature but also within neurons of brain regions affected in neurodegenerative diseases, such as the dorsal motor nucleus of the vagus, substantia nigra, and neocortex,^61^ underscoring the importance of investigating central nervous system involvement in FD beyond cerebrovascular lesions.

In the peripheral nervous system, pain in FD is thought to result from Gb3-induced degeneration of nerve fibers in the dorsal root ganglion neurons via TRPV channels and K^+^, Ca^2+^, and Na^+^ channels.^62^ Sugimoto et al^63^ demonstrated that Gb3 provoked mechanical allodynia in mice by enhancing proNGF-p75NTR signaling. Additionally, neuropathic pain in FD is associated with neuroimmune alterations.^64^ Choconta et al^65^ found that FD mice exhibited increased phagocytic activity of macrophages in the myeloid cell population of the dorsal root ganglion, suggesting macrophage involvement in the peripheral neuropathy of FD.

Conduction abnormalities in Aδ and C fibers are key contributors to autonomic neuropathy in FD. Aδ fibers transmit mechanical pain, while C fibers transmit temperature-related pain. Dysfunction of these fibers leads to autonomic disturbances, such as limb pain, abnormal sweating, and altered temperature sensation.^66^ However, once fiber damage reaches a critical threshold, these fibers can no longer transmit sufficient bioelectrical signals, which explains why older FD patients often experience less pain than younger individuals. Forstenpointner et al^67^ identified a correlation between fiber dysfunction and endothelial dysfunction, which are the two mechanisms underlying pain in FD patients, warranting further investigation for validation.

## Other organs

Cutaneous manifestations are often the earliest clinical signs in male FD patients, most notably the formation of angiokeratomas. Angiokeratomas are acquired benign vascular malformations that arise from Gb3 accumulation in dermal vascular endothelial cells and smooth muscle cells and dermal capillary dilation.^68^ Histologically, angiokeratomas are characterized by dilated subepidermal blood vessels, sometimes accompanied by epidermal acanthosis and hyperkeratosis. Clinically, angiokeratomas present as small, raised, dark red papules localized to the lower back, buttocks, groin, and upper thighs. These lesions serve as important markers for early recognition of FD.

Cutaneous lesions also significantly contribute to pain in FD. Üçeyler et al^69^ found that Gb3 accumulation in dermal fibroblasts impairs KCa1.1 activity and activates Notch1 signaling. Subsequently, by culturing keratinocytes and fibroblasts from FD patients, the team demonstrated that Gb3 deposition in skin fibroblasts increased KCa3.1 activity and IL-8 secretion, resulting in heightened sensitivity of cutaneous nociceptors.^70^ These findings confirm the involvement of the skin in pain in FD patients.

FD also affects the reproductive system, with reports of azoospermia and gonadal dysfunction.^71^^,^^72^ Sansone et al^73^ demonstrated that Gb3 accumulation in reproductive tissues impaired oxygen and nutrient delivery, leading to activation of hypoxia inducible factor 1 subunit alpha (HIF-1α), vascular endothelial growth factor A (VEGFA), and nuclear factor kappa B (NFκB). These alterations lead to cellular damage, thereby altering the seminiferous tubule microenvironment, reducing spermatogenesis, and ultimately resulting in infertility in male FD patients.

Ocular manifestations are also common in FD patients. Gb3 accumulation in ocular tissues can cause conjunctival and retinal vascular tortuosity, aneurysmal-like changes, cornea verticillata, and cataracts. Additional reported findings include impaired pupillary constriction, chronic uveitis, optic atrophy, visual field defects, and lacrimal gland involvement.^74^ Michaud et al^75^ demonstrated that insufficient oxygen perfusion to the optic nerve, retinal injury, and alterations in the retinal capillary plexus lead to retinal ganglion cell dysfunction. Yanık et al^76^ identified a potential interaction between choroidal and retinal microvasculature in FD patients, although the precise mechanisms remain to be elucidated. Similarly, auditory manifestations in FD, such as hearing loss, sudden deafness, and tinnitus, lack a clear pathophysiological explanation but may involve Gb3-induced vascular damage, thereby impairing cochlear blood supply.^77^ Collectively, these observations underscore the urgent need for further investigation into the pathophysiological mechanisms underlying ocular and auditory injury in FD.

## Overview and developments of the therapies in Fabry disease

**Figure 6:**
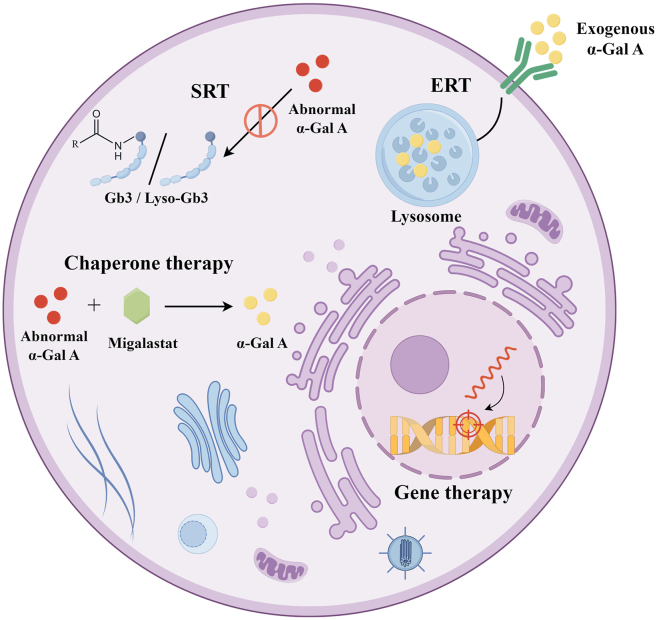
Principal therapeutic approaches in Fabry disease. Enzyme replacement therapy (ERT) involves regular intravenous administration of recombinant α-Gal A to reduce intracellular Gb3 and Lyso-Gb3 accumulation. Chaperone therapy can correct protein misfolding, stabilize the mutant enzyme within the endoplasmic reticulum and lysosomes, and partially restore catalytic activity. Gene therapy can deliver repaired, replaced, or edited genes into the human body via vectors to treat diseases. Substrate reduction therapy (SRT) aims to slow down disease progression by inhibiting glycosphingolipid synthesis, thereby reducing substrate accumulation. This figure is drawn by Figdraw (www.figdraw.com).

Currently, several modalities exist for FD management, including ERT and chaperone therapy, both of which aim to provide functional α-Gal A to reduce Gb3 accumulation, thereby restoring lysosomal trafficking and augmenting enzyme activity. In recent years, novel treatments have emerged, such as gene therapy and substrate reduction therapy, offering new avenues for FD management. Figure 6 depicts the principal therapeutic approaches currently employed in FD.

## Enzyme replacement therapy

ERT involves regular intravenous administration of recombinant α-Gal A to reduce intracellular Gb3 and Lyso-Gb3 accumulation. ERT can stabilize renal function, delay the progression to end-stage renal failure, improve myocardial function, decrease left ventricular hypertrophy, and alleviate acroparesthesia and hypohidrosis, thereby protecting renal, cardiac, and neurologic systems.^78^ Currently available ERT formulations include agalsidase α, which is produced in human fibroblasts (0.2 mg/kg every two weeks), and agalsidase β, which is produced in hamster ovary cells (1.0 mg/kg every two weeks). Both agalsidase α and β have received approval in multiple regions worldwide, although agalsidase α has not yet been approved by the US FDA.^79^

Although ERT has been widely used, several limitations persist. Firstly, ERT cannot completely clear the accumulated metabolic substrate, and it is ineffective against dysregulated cellular signaling, impaired autophagy, complement activation, and fibrosis. This explains why some patients exhibit a limited response to ERT.^48^^,^^57^^,^^80^ More importantly, as an exogenous therapy, ERT is inherently immunogenic. Over time, some patients develop antibodies that inhibit its efficacy.^81^ Additionally, infusion-related adverse events, the high treatment costs, and the burden of lifelong intravenous treatment negatively impact patients' quality of life. Hallows et al^82^ identified GLAv05 and GLAv09 variants that reduced the immunogenicity risk associated with ERT. Further research is needed to develop safer, more practical ERT formulations to address these shortcomings.

## Chaperone therapy

In some FD patients, the type of *GLA* gene mutation is a missense mutation, which results in misfolded proteins that undergo premature degradation, leading to reduced α-Gal A activity. Molecular chaperones assist in correcting protein misfolding, stabilizing the mutant enzyme within the endoplasmic reticulum and lysosomes, and partially restoring catalytic activity.^83^ Migalastat is currently the most commonly used medication in chaperone therapy.^84^ Compared with ERT, migalastat offers several advantages. Firstly, it is non-immunogenic, thus avoiding antibody-related immune responses. Moreover, it is administered orally, eliminating the need for intravenous infusions. Additionally, it better mimics the function of the endogenous enzyme, improving its distribution in tissues and cells as well as its ability to cross the blood–brain barrier. Overall, chaperone therapy for FD is relatively safe and effective, though its applicability is limited to certain mutation types.

## Gene therapy

Gene therapy can deliver repaired, replaced, or edited genes into the human body via vectors to treat diseases. As a monogenic disorder, FD is an ideal candidate for gene therapy. The efficacy of gene therapy depends largely on the choice of vectors. Among viral vectors, several have been developed that efficiently express target genes in cells, such as adenoviruses, adeno-associated viruses, and lentiviruses. These vectors can directly correct gene defects and provide sustained effects.^39^ Non-viral vectors are not limited by the size of the genetic material but have lower transfection efficiency, which remains a challenge to overcome.^85^

In the past five years, multiple studies related to gene therapy for FD have been reported,^86^, ^87^, ^88^, ^89^, ^90^, ^91^, ^92^, ^93^, ^94^, ^95^ with most focusing on exploring new effective vectors. For instance, Pagant et al^95^ demonstrated that *in vivo* gene editing mediated by zinc finger nucleases (ZFN) in the liver of Fabry mice led to increased α-Gal A activity in plasma and tissues, providing preclinical evidence for ZFN-mediated *in vivo* gene editing. Gene therapy may be an effective treatment option for FD, but further research, especially clinical studies, is needed to verify its efficacy and safety.

## Substrate reduction therapy

Substrate reduction therapy aims to slow down disease progression by inhibiting glycosphingolipid synthesis, thereby reducing substrate accumulation. Glucosylceramide synthase inhibitors are the main drugs of substrate reduction therapy, including Venglustat and Lucerastat, which reduce the production of substrates such as Gb3 by inhibiting the enzymatic reaction between ceramide and glucose. For instance, a phase Ⅱ clinical study by Deegan et al^96^ demonstrated that oral Venglustat rapidly reduced proximal markers (GL-1 and GM3) in the glycosphingolipid synthesis pathway and gradually decreased accumulation of Gb3 and Lyso-Gb3. Although both drugs have shown acceptable efficacy and tolerability, their ability to cross the blood–brain barrier poses potential risks of neurological and psychiatric adverse effects. AL01211, a non-brain-penetrant glucosylceramide synthase inhibitor with minimal central nervous system penetration, significantly reduces the incidence of neurological and psychiatric adverse events. A phase Ⅰ clinical trial has confirmed the efficacy and favorable tolerability of AL01211,^97^ but its findings require further evaluation in larger-scale studies.

In recent years, small interfering RNA (siRNA)-based therapy has emerged as a promising approach for substrate reduction therapy. The effectiveness of siRNA therapy depends on delivery systems capable of protecting nucleic acids and promoting their interaction with target cells. Lipid nanoparticles have been utilized as carriers for siRNA delivery. Beraza-Millor et al^98^ proposed a novel nanomedicine using lipid nanoparticles as siRNA delivery vectors. This approach enables substrate elimination through targeted silencing therapy and holds potential for the treatment of FD.

## Other therapies

Some studies have aimed to alleviate pathological damage in FD through unconventional approaches. Ceria-Zirconia nanoparticles have been shown to promote Gb3 metabolism by modulating cellular autophagy, thereby mitigating organ injuries in FD.^99^ This finding provides a foundation for the development of novel pharmacological therapies for FD. Apabetalone, an oral bromodomain and extra-terminal protein inhibitor (BETi), has been found to control the progression of FD by reducing inflammation and oxidative stress,^100^ but its therapeutic efficacy still requires further evaluation in clinical settings.

## Conclusions and perspectives

As a multi-organ disease, FD exhibits significant clinical heterogeneity, and current diagnostic approaches and treatment remain limited, leading to prolonged organ damage, which would impair patient prognosis and quality of life. Therefore, there is an urgent need to develop novel diagnostic tools and therapeutic strategies for FD. A comprehensive understanding of the pathophysiological mechanisms underlying target organ involvement plays a pivotal role in advancing early diagnosis and treatment. As an emerging tool, omics provide novel perspectives for advancing the understanding of the pathophysiological mechanisms, identifying novel biomarkers, and finding potential therapeutic targets of FD. This review summarizes the current understanding of FD pathophysiology and therapeutic approaches, with a focus on recent progress in mechanisms of target organs' involvement (particularly the kidneys and heart) through omics-based analyses. However, translating omics-based findings from bench to bedside faces several challenges. Current omics studies in FD often involve small and heterogeneous patient cohorts, so many proposed biomarkers require validation in larger, multicenter populations. Additionally, integrating multi-omics data and distinguishing clinically meaningful signals from background noise remain significant hurdles. Despite these limitations, continued advancements in high-throughput technologies and bioinformatics, coupled with collaborative research, hold promise for overcoming these barriers. Future studies should focus on confirming the clinical utility of candidate biomarkers and therapeutic targets, as well as addressing the cost and accessibility of omics-based tests. By addressing these challenges, we can accelerate the translation of omics discoveries into improved early diagnostic tools, better disease monitoring, and personalized therapeutic strategies for FD.

## CRediT authorship contribution statement

**Zhiyuan Wei:** Writing – original draft. **Junlan Yang:** Writing – review & editing. **Zhongyu Han:** Software. **Xiaoliang Zhang:** Writing – review & editing. **Bin Wang:** Writing – review & editing.

## Funding

This work was supported by Young Scholars of 10.13039/501100005240Yangtze River Scholar Professor Program (China) (No. 2023 BW); 10.13039/501100013058Jiangsu Provincial Key Research and Development Program (China) (No. BE2023770); Research Personnel Cultivation Programme of Zhongda Hospital Southeast University (China) (No. CZXM-GSP-RC150); the Fundamental Research Funds for the Central Universities (China) (No. 2242024k30041); Jiangsu Province High-Level Hospital Construction Funds of Zhongda Hospital, School of Medicine, 10.13039/501100008081Southeast University (China) (No. GSP-JCYJ-01); and Zhongda Hospital Affiliated to 10.13039/501100008081Southeast University, 10.13039/501100002949Jiangsu Province High-Level Hospital Construction Funds (China) (No. GSP-LCYJFH08).

## Conflict of interests

All the authors declared no competing interests.
